# Let-7 MicroRNA Family Is Selectively Secreted into the Extracellular Environment via Exosomes in a Metastatic Gastric Cancer Cell Line

**DOI:** 10.1371/journal.pone.0013247

**Published:** 2010-10-08

**Authors:** Keiichi Ohshima, Kanako Inoue, Akemi Fujiwara, Keiichi Hatakeyama, Kaori Kanto, Yuko Watanabe, Koji Muramatsu, Yorikane Fukuda, Shun-ichiro Ogura, Ken Yamaguchi, Tohru Mochizuki

**Affiliations:** 1 Medical Genetics Division, Shizuoka Cancer Center Research Institute, Shizuoka, Japan; 2 Pathology Division, Shizuoka Cancer Center Hospital, Shizuoka, Japan; 3 Division of Biotechnology and Life Science, Graduate School of Engineering, Tokyo University of Agriculture and Technology, Tokyo, Japan; 4 Shizuoka Cancer Center Hospital and Research Institute, Shizuoka, Japan; Universität Heidelberg, Germany

## Abstract

**Background:**

Exosomes play a major role in cell-to-cell communication, targeting cells to transfer exosomal molecules including proteins, mRNAs, and microRNAs (miRNAs) by an endocytosis-like pathway. miRNAs are small noncoding RNA molecules on average 22 nucleotides in length that regulate numerous biological processes including cancer pathogenesis and mediate gene down-regulation by targeting mRNAs to induce RNA degradation and/or interfering with translation. Recent reports imply that miRNAs can be stably detected in circulating plasma and serum since miRNAs are packaged by exosomes to be protected from RNA degradation. Thus, profiling exosomal miRNAs are in need to clarify intercellular signaling and discover a novel disease marker as well.

**Methodology/Principal Findings:**

Exosomes were isolated from cultured cancer cell lines and their quality was validated by analyses of transmission electron microscopy and western blotting. One of the cell lines tested, a metastatic gastric cancer cell line, AZ-P7a, showed the highest RNA yield in the released exosomes and distinctive shape in morphology. In addition, RNAs were isolated from cells and culture media, and profiles of these three miRNA fractions were obtained using microarray analysis. By comparing signal intensities of microarray data and the following validation using RT-PCR analysis, we found that let-7 miRNA family was abundant in both the intracellular and extracellular fractions from AZ-P7a cells, while low metastatic AZ-521, the parental cell line of AZ-P7a, as well as other cancer cell lines showed no such propensity.

**Conclusions/Significance:**

The enrichment of let-7 miRNA family in the extracellular fractions, particularly, in the exosomes from AZ-P7a cells may reflect their oncogenic characteristics including tumorigenesis and metastasis. Since let-7 miRNAs generally play a tumor-suppressive role as targeting oncogenes such as *RAS* and *HMGA2*, our results suggest that AZ-P7a cells release let-7 miRNAs via exosomes into the extracellular environment to maintain their oncogenesis.

## Introduction

Cells secrete different types of small membrane vesicles. Exosomes are one of the vesicles with 30–100 nm diameter and physicochemically distinct from other secreted vesicles [1]. Inside exosomes, cellular gene products including proteins, mRNAs, and microRNAs (miRNAs) are packaged and these molecules can be transferred to recipient cells to deliver their molecular signaling such as oncogenesis [2]–[4] and immune response [1], [5]. Exosomes are released by many types of cells [6] which include tumor cells, epithelial cells, and hematopoietic cells such as reticulocytes, cytotoxic T lymphocytes, Epstein-Barr virus-transformed B cells, mastocytes, dendritic cells, and platelets. Secreted exosomes have been isolated and characterized *in vitro* from cultured cell lines [7] along with *in vivo* in body fluids [7] including blood [8], urine [9], saliva [10], amniotic fluid [11], and malignant pleural effusions [12]. Since observed in many proliferating cell types, it is conceivable to exacerbate tumor cells, as evidenced by their increased presence in plasma and pleural effusions of patients with cancer [8], [12]. This increased presence in non-invasive body fluids of cancer patients has accelerated to profile molecular components in the exosomes for discovering clinically useful tumor markers and biomarkers [3], [7], [13].

miRNAs are a class of noncoding small RNAs that are involved in post-translational regulation of gene expression by inhibiting both stability and translation of mRNAs [14]. Recent evidence has shown that miRNA mutations or misexpression correlate with various human cancers and indicate that some miRNAs can function as oncogenes or tumor suppressors [15], [16]. To analyze RNAs, it is always to consider their stability from degradation by RNase. Recent findings indicate that endogenous plasma miRNAs in blood samples are stably detectable in a form that is resistant to RNase activity [17], evidenced by identification of miRNAs in body fluids such as blood [17]–[24], urine [25], and saliva [10], [26].

Cultured cancer cells have been used to search for tumor markers. In particular, identifying proteins and peptides secreted into the culture media has developed by proteomics-based approach [27], [28]. As for molecular signature in the exosomes, proteomics as well as transcriptomics analyses have been performed to reveal tumorigenesis and identify tumor marker candidates [2]–[4], [7], [29]. Here, to identify miRNA related to tumorigenesis and metastasis, we performed extensive miRNA analysis in three cellular fractions including cells, exosomes, and medium from cultured cells. Ranking data of these intracellular and extracellular miRNAs obtained by microarray analysis, we found that let-7 miRNA family is rich in all the fractions from AZ-P7a cells, a metastatic gastric cancer cell line, which produces homogeneous and condensed morphology, and high recovery rate of exosomal miRNAs. These findings were distinct from other cell lines including lung cancer cell lines (SBC-3, NCI-H69, and DMS53), colorectal cancer cell lines (SW480 and SW620), and AZ-521, the parental cell line of AZ-P7a. Considering that let-7 miRNA family functions mainly as tumor suppressor genes [30] to target oncogenes such as *RAS* and high mobility group A2 (*HMGA2*) [31], we propose that AZ-P7a cells selectively secrete let-7 miRNAs into the extracellular environment via exosomes to maintain their tumorigenic and metastatic propensities.

## Results

### Isolation of exosomes from various cancer cell lines

**Figure 1:**
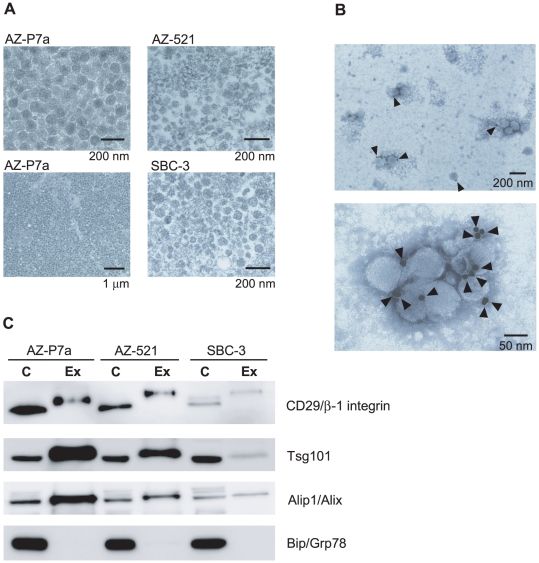
Characterization of exosomes. **A.** Morphological characterization of exosomes derived from AZ-P7a, AZ-521, and SBC-3 cells by transmission electron microscopy. **B.** Immunoelectron micrographs of AZ-P7a exosomes labeled with immunogold anti-CD63. Exosomes with black colloidal gold particles on the capsular membranes were observed as positive CD 63 staining under the transmission electron microscope (arrowheads). **C.** Molecular characterization of exosomes derived from AZ-P7a, AZ-521, and SBC-3 cells by western blot analysis. Protein extracts (10 µg) prepared from cells (C) or exosomes (Ex) were assessed using antibodies against exosomal protein markers (CD29/β1-integrin, Aip1/Alix, and Tsg101) and an endoplasmic reticulum marker (Bip/Grp78).

Exosomes are produced from inner cells to the extracellular environment via an exocytosis-like pathway [1]. In addition to body fluids such as serum and plasma from peripheral blood [7], [8], exosomes are found in the medium of cultured cells [7], facilitating identification of exosomal molecules including proteins, mRNAs, and miRNAs for the aim for their clinical use. Profiling such exosomal molecules produced in cultured cancer cells have led to discover a novel candidate marker and antigen for cancer diagnostics and immunotherapy [7]. In this study, for the purpose of profiling exosomal miRNAs, we first isolated exosomes from culture media of 46 cancer cell lines with various tissue types, which include 8 for stomach, 16 for lung, 5 for colon, 9 for pancreas, 3 for prostate, and 5 for breast. After cells were grown for 48 h, exosomes were collected with a combination of successive centrifugation and molecular weight cut-off membranes as described in Materials and Methods. Their purity of exosomal fractions was assessed by analyses of transmission electron microscopy and western blotting. Transmission electron microscopy showed round-shaped vesicular membrane structures approximately within the size of 100 nm in diameter. Among three cell lines, SBC-3, AZ-521, and AZ-P7a, it is of interest that the morphology from AZ-P7a cells was more homogeneous and condensed than other cells (Figure 1A). Immunoelectron microscopy showed that the extracellular particles isolated from the culture medium of AZ-P7a cells had immunoreactivity with an antibody against CD63, one of the exosomal membrane proteins, on the capsular membranes (Figure 1B). The presence of known exosomal proteins including CD29/β1-integrin, Aip1/Alix and tumor susceptibility gene 101 (Tsg101) was confirmed by western blot analysis, while a protein localized to endoplasmic reticulum, Bip/Grp78, was undetectable (Figure 1C). This result indicates that the contamination of material derived from other cellular compartments in the exosomal fractions was minimal. The yield of exosomal proteins from AZ-P7a cells was 10 times higher than that from AZ-521 cells; ∼2.5 mg proteins were obtained from 1×10^8^ AZ-P7a cells.

### Enrichment of exosomal RNAs derived from AZ-P7a cells

**Figure 2:**
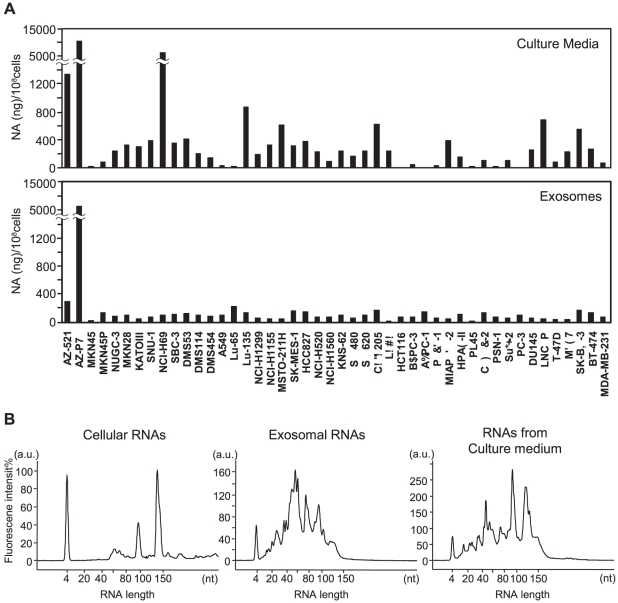
Total RNAs in exosomes and culture media from various cancer cell lines. **A.** Amounts of total RNAs recovered from exosomes (lower panel) and culture media (upper panel). The amount of total RNAs per cell was shown. **B.** Distribution in length of RNA. Isolated total RNAs from the exosomes and culture medium of AZ-P7a cells were detected using Bioanalyzer. The result of cellular total RNAs was shown for comparison.

After isolation, exosomal total RNAs were isolated from 46 cultured cell lines. Interestingly, the RNA yields from AZ-P7a cells were much greater than those from other cells (Figure 2A). The distribution in length showed the presence of large amounts of small RNAs in exosomes (center panel, Figure 2B). It should be noted that there is a significant difference in total exosomes and the exosomal miRNA levels between patients with lung adenocarcinoma and controls [8]. AZ-P7a cell line was established by repeated inoculation of the parental AZ-521 cells in mice, resulted in high peritoneal-metastatic potential [32], [33]. Thus, the high yields of both RNA and protein along with the morphological characteristics in AZ-P7a cells may be attributed to their high tumorigenic and metastatic propensities. Since miRNAs have been detected in cell-free body fluids [17]–[20], we also performed direct isolation of total RNAs from culture medium without procedure for isolating exosomes. The amounts of total RNAs from culture media were generally greater than those from exosomes (Figure 2A). RNA distribution in length showed that miRNA fractions were detected in the range from 10 to 40 nucleotide length in RNAs prepared from culture medium (right panel) as well as exosomes (center panel) from AZ-P7a cells (Figure 2B). According to the manufacture's protocol for Bioanalyzer, peaks with longer than 40 nucleotide length contain other small RNAs including tRNAs at 70∼90, 5S ribosomal RNA at 100, and 5.8S ribosomal RNA at 150, as observed in the intracellular fraction (left panel). These results suggest that miRNAs may exist in the culture medium outside the exosomal fraction although RNAs are believed to be protected from RNases as being packaged in exosomes. Particularly, compared to adherent cells, the increment of RNA yields was remarkably greater in floating cells (NCI-H69 and Lu-135) or cells with mix populations of floating and adherent cells (Colo205), which may be reflected by contamination of RNAs shed from lysed cells that have been continuously left in culture media.

### miRNA profiling by microarray analysis

**Figure 3:**
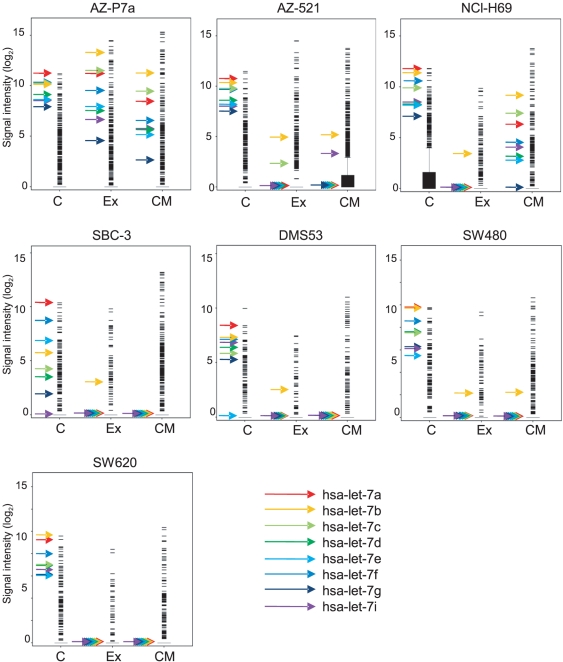
miRNA profiling in the intra- and extra-cellular fractions from AZ-P7a, AZ-521, NCI-H69, SBC-3, DMS53, SW480 and SW620 cells. miRNA profiles in cells (C), exosomes (Ex), and culture media (CM) were obtained by miRNA microarray analysis. Y axis represents log_2_ of hybridization signals, shown by bars and filled boxes. The arrows on the left side from the bunch of signals represent signals corresponding to let-7 miRNA family including let-7a (red), let-7b (yellow), let-7c (light green), let-7d (dark green), let-7e (sky blue), let-7f (blue), let-7g (navy blue), and let-7i (purple).

miRNA profiles in the intracellular and extracellular fractions were determined by microarray analysis for seven cancer cell lines including AZ-521, AZ-P7a, SBC-3, NCI-H69, DMS53, SW480, and SW620. Samples from culture media as well as exosomes provided hybridization signals (Figure 3), indicating the existence of miRNAs in these samples. Up to the present, analyzing microarray data for miRNA among samples, no clear methods have been made for normalization of signal intensity since it is difficult to find an appropriate internal standard material [34]. Thus, miRNA microarray data have been in general analyzed without normalization, assuming that the total amounts of input RNAs are constant among samples [34], [35]. In this study, after averaging miRNA microarray data between two replicates, we ranked miRNAs according to their signal intensities. We found that let-7 miRNA family such as let-7a, let-7b, let-7c, let-7d, let-7e, let-7f, let-7g, and let-7i were detected with relatively high signals throughout most of the intracellular fractions from the seven cell lines (Table 1, Figure 3). However, no or little signal intensities for the let-7 miRNAs were detected in the extracellular fractions except both extracellular fractions from AZ-P7a cells and culture medium fraction from NCI-H69 cells (Table 1, Figure 3). Particularly, in both the extracellular fractions from AZ-P7a cells, all the eight let-7 miRNAs were detected although the rank of let-7g was lower than other seven let-7 miRNAs. Based on the distinct patterns of let-7 miRNA levels among the three cellular fractions, we divided the seven cell lines into three groups (Figure 3) as follows; (1) AZ-P7a, cells that let-7 miRNAs were found in all the three fractions, (2) AZ-521 along with SBC-3, DMS53, SW480, and SW620, cells that let-7 miRNAs were generally found in only intracellular fractions, (3) NCI-H69, cells that let-7 miRNAs were found in the intracellular fractions as well as in the culture medium to some extent.

**Table 1:** Ranking of let-7 miRNA family in the intra- and extra-cellular fractions based on signal intensities from microarray data.

Cell line	Sample	Rank by signal intensity								
		let-7a	let-7b	let-7c	let-7d	let-7e	let-7f	let-7g	let-7i	miRNAs
AZ-P7a	Cells	2	9	8	14	20	6	29	22	202
	Ex	10	4	9	34	28	15	90	51	212
	CM	30	13	22	71	83	46	147	63	209
AZ-521	Cells	3	4	5	13	17	6	22	18	171
	Ex	–	53	130	–	–	–	–	–	153
	CM	–	99	–	–	–	–	–	158	225
NCI-H69	Cells	1	2	6	25	27	4	44	23	261
	Ex	–	41	–	–	–	–	–	–	102
	CM	36	16	24	86	91	55	–	69	131
SBC-3	Cells	1	31	56	83	17	7	115	–	132
	Ex	–	38	–	–	–	–	–	–	56
	CM	–	–	–	–	–	–	–	–	186
DMS53	Cells	2	8	20	16	–	9	29	10	85
	Ex	–	25	–	–	–	–	–	–	42
	CM	–	–	–	–	–	–	–	–	86
SW480	Cells	1	2	15	13	36	6	23	25	124
	Ex	–	33	–	–	–	–	–	–	47
	CM	–	72	–	–	–	–	–	–	106
SW620	Cells	2	1	13	14	23	7	21	16	107
	Ex	–	–	–	–	–	–	–	–	32
	CM	–	–	–	–	–	–	–	–	88

Ex = exosomes. CM = culture medium. miRNAs = the total number of miRNAs detected. A dash (-) indicates no detection.

### RT-PCR analyses of intracellular and extracellular let-7 miRNA family

**Figure 4:**
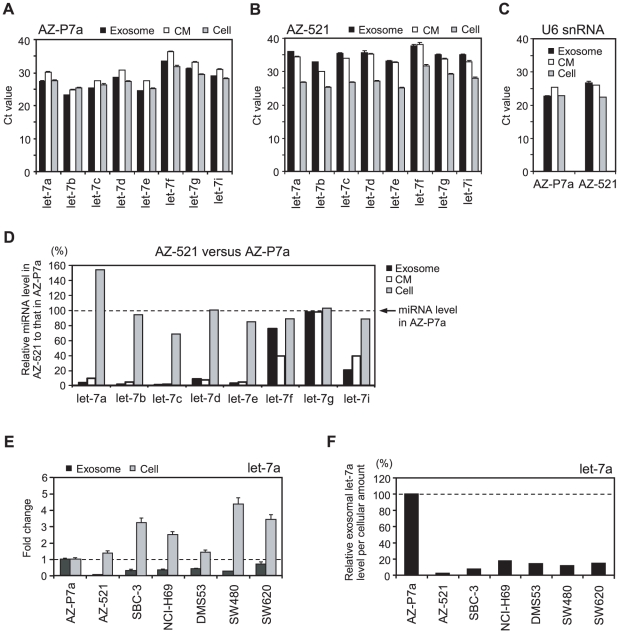
RT-PCR analyses of intra- and extra-cellular let-7 miRNA family. **A**, **B.** Levels of mature let-7 miRNA family including let-7a, let-7b, let-7c, let-7d, let-7e, let-7f, let-7g, and let-7i in cells (shaded bars), exosomes (filled bars), and culture medium (open bars) from AZ-P7a cells (A) and AZ-521 cells (B). Each miRNA level was analyzed using quantitative RT-PCR. The cycle threshold (Ct) value is presented as the mean ± SD (n = 4). **C.** Levels of U6 snRNA in cells (shaded bars), exosomes (filled bars), and culture medium (open bars) from AZ-P7a and AZ-521 cells detected by quantitative RT-PCR analysis. The cycle threshold (Ct) value is presented as the mean ± SD (n = 4). **D.** Relative amounts of let-7 miRNA family in cells (shaded bars), exosomes (filled bars), and culture medium (open bars) from AZ-521 cells versus AZ-P7a cells. The dotted line and arrow represents the levels of let-7 miRNA family in AZ-P7a cells, shown as 100%. The levels of U6 snRNA in each sample were used as an internal standard for normalization amounts of let-7 miRNAs. Except let-7f and let-7g, the levels of let-7 miRNAs were reduced in the extracellular fractions. **E.** Relative amounts of let-7a in cells (shaded bars) and exosomes (filled bars) from 7 cancer cell lines including AZ-P7a, AZ-521, NCI-H69, SBC-3, DMS53, SW480 and SW620 cells. The dotted line represents the levels in AZ-P7a cells, shown as 1. The levels of U6 snRNA in each sample were used for normalized amounts of let-7a. The value of fold change is presented as the mean ± SD (n = 3) from samples independently prepared from cell culture. **F.** Relative amounts of exosomal let-7a in 7 cancer cell lines. The dotted line represents the level in AZ-P7a cells, shown as 100%.

**Figure 5:**
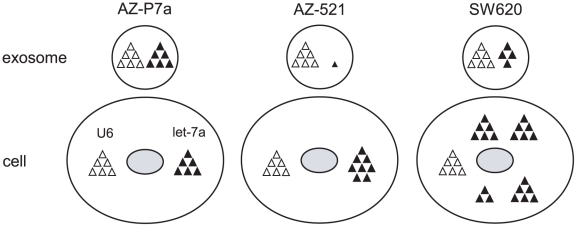
Models on difference in localization of let-7 miRNA family. Based on the results obtained by microarray and RT-PCR analyses, these models were drawn. In comparison between AZ-P7a cells (left) and AZ-521cells (center), normalized by the amount of U6 snRNA (open triangles), the amount of exosomal let-7a miRNA (filled triangles) in AZ-521 cells was approximately 3% of that in AZ-P7a cells while the intracellular amount in AZ-521 cells was rather 1.4 times greater than that in AZ-P7a cells (Figure 4E). These models are applied to six of eight let-7 miRNA family tested, including let-7a, let-7b, let-7c, let-7d, let-7e, and let-7i. In SW620 cells (right), the amount of let-7a was 3.5 times greater than that in AZ-P7a cells while the amount of exosomal let-7a was 0.7 times less than in AZ-P7a cells (Figure 4E).

We then performed quantitative RT-PCR for the eight let-7 miRNA family to validate the microarray data. let-7 miRNAs were readily detected in the intracellular and extracellular fractions from AZ-P7a cells (Figure 4A) and AZ-521 cells (Figure 4B). The levels of let-7 miRNAs per input RNAs were generally higher in the extracellular fractions from AZ-P7a cells than from AZ-521 cells. For normalizing the levels of target miRNAs obtained by RT-PCR, U6 snRNA has been generally used as an internal control. We observed the presence of U6 snRNA in all the three fractions from AZ-P7a cells and AZ-521 cells (Figure 4C) and then compared the levels of let-7 miRNAs between the corresponding fractions from these two cell lines. After normalization, the levels of let-7 miRNAs including let-7a, let-7b, let-7c, let-7d, let-7e, and let-7i in both the fractions of exosomes and culture media from AZ-521 cells were lowered as compared with those from AZ-P7a cells. On the contrary, there was no large difference in the levels of the intracellular let-7 miRNAs. We next compared the level of exosomal let-7a from AZ-P7a cells with those from other cancer cell lines including SBC-3, NCI-H69, DMS53, SW480, and SW620, and found that the let-7a level only from SW620 cells was relatively close (0.7 times) to the level from AZ-P7a cells (Figure 4E). It should be noticed that the intracellular level of let-7a from SW620 cells was approximately 3.5 times more abundant than that from AZ-P7a cells. Based on these results, we assumed localization of let-7a in the fractions of cells and exosomes (Figure 5), and conducted further analysis. Normalized by the U6 levels, we calculated relative levels of let-7a packaged in exosomes per the levels of cellular let-7a (Figure 4F). As a result, the amount of exosomal let-7a was much greater in AZ-P7a cells than other six cells, suggesting that AZ-P7a cells were selectively and actively secreted let-7 miRNA family into the extracellular environment via exosomes.

## Discussion

In the present study, we revealed that let-7 miRNA family was secreted into the extracellular environment via exosomal transport, which was specific to a metastatic gastric cancer cell line, AZ-P7a. let-7 miRNA family in human consists of 10 sequences from 13 precursors [30]. In general, let-7 miRNAs act as a tumor suppressor by targeting oncogenes such as *RAS* and *HMGA2* and let-7 miRNAs are downregulated in many cancers from solid organs [31]. AZ-P7a cells possess a metastatic ability with peritoneal dissemination in nude mice [32], [33]. Thus, we propose that the exosomal release of let-7 miRNAs into the extracellular environment results in decrease of anti-tumorigenic effect within the cells, which lead to maintain their oncogenesis and invasiveness. On the other hand, let-7 miRNAs less frequently play an oncogenic function, due to increase of expression by hypomethylation at the let-7 locus [36] or targeting caspase-3 mRNA [37]. In this case, the exosomal release of let-7 miRNAs may cause transformation in target cells by transferring their oncogenic properties.

In patients with lung cancer, the total levels of exosomes and their miRNAs increase compared to controls [8]. It has been demonstrated that tumor exosomes induce immune escape mechanism in cancers by spontaneous T cell apoptosis [38]–[40]. In this study, we have shown homogeneous morphology and enrichment of RNAs in exosomes from metastatic AZ-P7a cells. This may be reflected by their tumorigenicity.

miRNAs are very stable in blood plasma and serum since protected from RNases [17]. Thus, it makes miRNA levels tested for clinical diagnosis as tumor markers and biomarkers. How does it happen? Chin et al. have suggested two hypotheses for the source of miRNAs on circulating blood samples; it may be due to a result of tumor cell death and lyses or release by tumor cells into the extracellular microenvironment of blood vessels [19]. On the contrary to adherent cells, we observed that the amounts of total RNAs in culture media were relatively higher than in exosomes for cells with floating property such as NCI-H69, Lu-135, and Colo205. This increment probably resulted in the contamination of RNAs from dead cells in culture media.

For the discovery of novel blood tumor markers and biomarkers, omics approaches including proteomics and transcriptomics are conducted either by direct analysis of blood samples or application of profiling in tissue samples. There are contradictory reports if whether the profiles of miRNA and mRNAs in bloodstream are parallel with the tumor's profile. The signature of exosomal miRNAs reflects that of the originating tumor cells in patients with lung adenocarcinoma [8] and breast cancer [41]. On the other hand, Skog et al. have shown that microarray analysis for mRNA derived from glioblastoma cells and the corresponding exosomes revealed mRNAs exclusively detected in microvesicles, speculating that mRNAs are localized to a specific region of cytoplasm [3]. In addition, Tanaka et al. have demonstrated that the level of miR-92a decreased in the blood plasma of acute leukemia patients while its strong expression was found in the tissue samples [24]. Our results on selective enrichment of let-7 miRNA family in AZ-P7a cells-derived exosomes fit with latter case. Unless target molecules are miRNAs derived from circulating tumor cells, it suggests careful investigation are needed to use miRNA profiles in tissue samples for detection in serum or plasma samples.

Since exosomes are produced in hematopoietic cells as well as epithelial cells, circulating blood contains exosomes derived from both type of cells. In general, to investigate exosomal profiles of proteins, mRNAs, and miRNAs in serum and plasma, exosomes derived from tumors with epithelial characteristics are separated from those from hematopoietic cells [8], [13]. This is performed using affinity purification with antibodies against molecules such as EpCAM that express on the surface of epithelial cells. Our findings that there were similar levels of let-7 miRNA family between exosomes and culture media from AZ-P7a cells suggest that these miRNA levels may be measured without isolating exosomes from clinical samples such as serum and plasma.

In conclusion, this study demonstrated that let-7 miRNA family is rich in exosomes from a metastatic gastric cancer cell line, AZ-P7a cells. Since let-7 miRNAs are involved in proliferation process [31], their exosomal secretion may play an important role in tumorigenesis and metastasis.

## Materials and Methods

### Cell culture

Human stomach cancer cell lines (AZ-521, MKN45, KATOIII, and NUGC-3), human lung cancer cell lines (SBC-3, Lu-65, Lu-135, and KNS-62), and human pancreatic cancer cells (Suit-2) were purchased from Japanese Collection of Research Bioresources. Human stomach cancer cells (SNU-1), human lung cancer cell lines (DMS-53, DMS114, NCI-H69, A549, NCI-H1299, NCI-H1155, NCI-H520, NCI-H1560, SK-MES-1, and HCC827), human mesothelioma cells (MSTO-H211), human colorectal cancer cell lines (SW480, SW620, Colo205, LoVo, and HCT116, human pancreatic cancer cell lines (BxPC-3, AsPC-1, Panc-1, MIAPaca-2, HPAF-II, PL45, and Capan-2), human prostate cancer cell lines (PC-3, SU145, and LNCaP), and human breast cancer cell lines (T-47D, McF7, SK-BR-3, BT-474, and MDA-MB-231) were purchased from American Type Culture Collection. MKN28 (human stomach cancer cells) and PSN-1 (human pancreatic cancer cells) were purchased from Immuno-Biological Laboratories and European Collection of Cell Culture, respectively. Human stomach cancer cell lines, AZ-P7a [32], [33] and MKN45P [42] were gift from Drs. Yutaka Yonemura and Yoshio Endo. In AZ-521 and AZ-P7a cells, no RT-PCR products were observed for 14 retroviruses including HPV16, EBV, CMV, SV40, HBV, HCV, BKV, JCV, HHV7, HTLV1, HTLV2, HIV1, HIV2, and ADV type 1 (data not shown), excluding infection by these retroviruses in both the cell lines. Cells were maintained in RPMI-1640 medium (Sigma-Aldrich) supplemented with 10% heat-inactivated fetal bovine serum (Invitrogen), 100 U/ml penicillin, and 0.1 mg/ml of streptomycin.

### Production and isolation of exosomes

Adherent cells were seeded to 150 mm-dishes at appropriate number of cells ranging from 3×10^6^ to 1×10^7^ cells per dish, while floating cells were inoculated into 75 cm^2^-flask at 2∼2.5×10^7^ cells per flask. Cells were cultured in complete RPMI-1640 medium of 25 ml and 50 ml for dishes and flasks, respectively, for 24 h at 37°C and 5% of CO_2_, washed twice with phosphate-buffered saline (PBS), and incubated 48 h in phenol red-free RPMI-1640 medium (Sigma-Aldrich) containing 10% heat-inactivated fetal bovine serum which was previously depleted contaminating microvesicles by overnight centrifugation at 100,000×g. Exosomes were isolated from the total cells ranging from 6×10^7^ to 3×10^8^ cells as previously described [29]. Briefly, culture medium was collected and centrifuged at 800×g for 5 min and additional 2,000×g for 10 min to remove lifted cells. The supernatant was subjected to filtration on a 0.1 µm pore polyethersulfone membrane filter (Corning) to remove cell debris and large vesicles, followed by concentration by a 100,000 Mw cut-off membrane (CentriPlus-70, Millipore). The volume of supernatant was reduced from approximately 250–500 ml to approximately 30 ml. The supernatant was then ultracentrifuged at 100,000×g for 1 h at 4°C using 70Ti rotor (Beckman Coulter). The resulting pellets were resuspended in 6 ml PBS and ultracentrifuged at 100,000×g for 1 h at 4°C using 100Ti rotor (Beckman Coulter).

### Transmission electron microscopy

The pelleted exosomes were mixed with equal quantities of freshly prepared 2% glutaraldehyde in PBS, incubated overnight at 4°C, postfixed with 1% osmium tetroxide in PBS at 4°C for 2 h, and dehydrated in a graded series of ethanol. Following dehydration, the samples were transferred to propylene oxide and embedded in epoxy resin Quetol 812 (Nisshin EM). Ultrathin sections were cut with Ultracut UCT (LEICA), stained with uranyl acetate and lead citrate, and observed with the JEM-1230 Electron Microscope (JEOL). Immunoelectron microscopic analysis was performed according to the method described by Xiao et al. [43] with minor modifications. In brief, exosomes were mixed and incubated with mouse anti-human CD63 monoclonal antibody (catalog no. sc-51662, Santa Cruz) for 1 h at room temperature. The sample was dropped onto the membrane surface of a copper mesh (collodion/carbon coated 400 mesh, Nisshin EM) and incubated for 1 h at room temperature. After wash with PBS, immunogold conjugated goat anti-mouse IgG (catalog no. EM.GMHL15, BBInternational) diluted at 1∶20 was dropped onto the membrane. The copper mesh was floated in the droplets with its membrane surface faced down at room temperature for 30 min. After wash with PBS, uranyl acetate drops was put onto the copper mesh surface and stained at room temperature for 30 s. Exosomes with black colloidal gold particles on the capsular membranes were marked as positive under the transmission electron microscope.

### Western blot analysis

Cells and exosomal fractions were washed, resuspended in a lysis buffer [7.5 M urea (Sigma-Aldrich), 2.5 M thiourea (Sigma-Aldrich), 12.5% glycerol (Wako), 50 mM Tris, 2.5% n-octyl-beta-D-glucoside (Sigma-Aldrich), 6.25 mM Tris(2-carboxyethyl)phosphine hydrochloride (Sigma-Aldrich), 1.25 mM protease inhibitor (catalog no. P2714, Sigma-Aldrich)], and incubated for 1 h at 4°C using the Rotator RT-50 (TAITEC). After centrifugation at 14,000×g for 60 min at 4°C, the supernatant was collected and protein concentration was determined by the Bradford Protein Assay Kit (Bio-Rad). A portion of proteins (10 µg) were denatured by boiling in Laemmli sample buffer, run on 10% SDS-polyacrylamide gels, and transferred to Immobilon-P PVDF membranes (0.45 µm pore size, Millipore). The blots were blocked for 1 h with 5% non-fat dry milk in Tris-buffered saline containing Tween 20 (10 mM Tris–HCl, pH 7.5, 150 mM NaCl and 0.01% Tween 20). After blocking the membranes were incubated with each primary antibody or antiserum including rabbit anti-Tsg101 serum (catalog no. T5951, Sigma-Aldrich), goat anti-Aip1/Alix serum (catalog no. sc-49268, Santa Cruz), mouse monoclonal anti-CD29/β1-integrin (catalog no. sc-610468, BD Biosciences), and mouse monoclonal anti-Bip/Grp78 (catalog no. 610979, BD Biosciences) at a dilution of 1∶200 for 1 h at room temperature. The blots were then incubated with the corresponding anti-IgG secondary antibody conjugated with horseradish peroxidase (Jackson Laboratories) at a dilution of 1∶2,000 for 1 h at room temperature. Specific proteins were visualized using the ECL system (GE Healthcare) and the FUJIFILM Luminescent Image Analyzer LAS3000 (Fuji Film). The protein molecular weight was deduced using the Precision Plus Protein Standards (Bio-Rad Laboratories).

### RNA Isolation

For RNA isolation from culture medium, cells were seeded as described above. 48 h after grown, culture medium was collected and centrifuged at 800×g for 5 min and additional 2,000×g for 10 min. The supernatant was subjected to filtration on a 0.22 µm pore polyethersulfone membrane filter (Corning), followed by concentration with a 5,000 Mw cut-off membrane (Centricon Plus-70, Millipore). The final volume of supernatant was approximately 10–20 ml from the original volume of 250–500 ml. The supernatant was mixed with equal volume of QIAzol Lysis Reagent (Qiagen) and followed the procedure for RNA isolation described below. Total RNA was isolated from cells, exosomes, or culture media using the miRNeasy Mini Kit (Qiagen) according to the manufacture's instructions. To remove genomic DNA, 40 µg of cellular total RNA were incubated with 40 units of RQ1 RNase-Free DNase (Promega) at 37°C for 30 min in the presence of 40 units of RNasin Plus RNase Inhibitor (Promega). For total RNA from exosomes and culture media, all amounts of crude products were treated in the same procedure except the use of 5 U DNase. RNA was purified using the RNeasy MinElute Cleanup Kit (Qiagen) according to the manufacturer's instructions. The RNA samples were quantified with a NanoDrop spectrophotometer (Thermo Fisher Scientific) and assessed using the Agilent Small RNA Kit and the Agilent 2100 Bioanalyzer (Agilent Technologies).

### Microarray analysis

For miRNA profiling, 100 ng and 20 ng of cellular and extracellular total RNAs, respectively, were fluorescence-labeled using the miRNA Complete Labeling Reagent and Hyb Kit (Agilent Technologies) according to manufacture's protocol (Agilent miRNA microarrays version 2.2). The comprehensive miRNA analysis was performed using the Human miRNA Microarray Kit (8×15 K) Ver.3.0 (Agilent). Hybridization signals were detected with the DNA Microarray Scanner (Agilent Technologies). The scanned images were analyzed using the Agilent Feature Extraction software and data analysis was performed using the GeneSpring GX software (Agilent Technologies). The data discussed in this manuscript have been deposited in NCBI's Gene Expression Omnibus (GEO) and are accessible through GEO Series accession number GSE21350: http://www.ncbi.nlm.nih.gov/geo/query/acc.cgi?acc=GSE21350).

### Reverse transcription (RT) and quantitative RT-PCR for miRNA analysis

Quantitative miRNA levels were determined using real-time RT-PCR with the Applied Biosystems 7900 HT Sequence Detection System (Applied Biosystems), TaqMan® Gene Expression Assay (Applied Biosystems) for human let-7a (assay ID 000377), let-7b (assay ID 002619), let-7c (assay ID 000379), let-7d (assay ID 002283), let-7e (assay ID 002406), let-7f (assay ID 000382), let-7g (assay ID 002282), let-7i (assay ID 002221), and U6 snRNA (assay ID 001973) as an endogenous control. Ten ng of total RNA were subjected to reverse transcription with TaqMan® Universal PCR Master Mix No AmpErase (Applied Biosystems) and the respective TaqMan® reagents for target genes. RT-PCR was carried out in a total volume of 20 µl reaction mixture according to the manufacture's protocol. Amplification was carried out as follows: 95°C for 10 min, 40 cycles of 95°C for 15 s and 60°C for 60 s. Samples were analyzed in triplicate as biological replicate or quadruplicate as technical replicate. The miRNA levels were defined from the cycle threshold (Ct), the comparative Ct method, and normalization by the level of U6 snRNA in each sample. Fold increases or decreases in each miRNA level of cell lines tested were determined by reference to the level of AZ-P7a cell line.
